# Metabolically Healthy Obesity and Metabolic Dysfunction-Associated Steatotic Liver Disease (MASLD): Navigating the Controversies in Disease Development and Progression

**DOI:** 10.1007/s13679-025-00637-9

**Published:** 2025-05-19

**Authors:** Chrysi Koliaki, Maria Dalamaga, Konstantinos Kakounis, Stavros Liatis

**Affiliations:** 1 First Propaedeutic Department of Internal Medicine and Diabetes Center, Medical School, Laiko General Hospital, National Kapodistrian University of Athens, 17 Agiou Thoma Street, Athens, 11527 Greece; 2 Department of Biologic Chemistry, Medical School, National Kapodistrian University of Athens, Athens, Greece; 3 Department of Gastroenterology, Hippokration General Hospital of Athens, Athens, Greece

**Keywords:** Adiposity, Metabolic dysfunction-associated steatotic liver disease, Obesity, Metabolic health, Metabolically healthy obesity, Metabolically unhealthy obesity, Obesity phenotypes

## Abstract

**Purpose of Review:**

The natural course of metabolic dysfunction-associated steatotic liver disease (MASLD) in the population with metabolically healthy obesity (MHO) has not been adequately explored. In the present narrative review, we summarize the evidence regarding the association between MHO and MASLD prevalence, incidence and progression.

**Recent Findings:**

Cross-sectional, population-based, cohort studies have shown an increased prevalence of hepatic steatosis and fibrosis in subjects with MHO compared with metabolically healthy non-obese individuals (MHNO). In large-scale longitudinal cohort studies among metabolically healthy subjects, increasing body mass index (BMI) has been found to be independently associated with an increased incidence of MASLD and progressive hepatic fibrosis over a mean follow-up period of 2.2–7.7 years. With regard to advanced MASLD, the prevalence of steatohepatitis and clinically significant liver fibrosis is lower in MHO compared with subjects with metabolically unhealthy obesity (MUO). The presence of MASLD has been proposed as a strong risk factor for metabolic health deterioration in MHO. Furthermore, subjects with MHO and MASLD display an elevated 10-year cardiovascular risk and a three-fold increased risk of incident diabetes compared with MHO without MASLD. MASLD may also predict the failure to convert from MUO to MHO after a weight loss intervention.

## Abstract (de)

Subjects with MHO have an increased prevalence of MASLD and hepatic fibrosis risk. We suggest that subjects with MHO and MASLD cannot be characterized as truly metabolically healthy, and we emphasize the need to incorporate hepatic health assessment into the MHO definition, to improve its predictive value for cardiometabolic outcomes.

## Introduction

Metabolic dysfunction-associated steatotic liver disease (MASLD) is currently the most common chronic liver disease [1, 2]. Its overall global prevalence has steadily increased during the past decades and is now estimated at 38% [3, 4]. MASLD is a systemic metabolic disease with serious hepatic and extrahepatic complications [5]. Approximately 10–30% of patients with hepatic steatosis are expected to progress to metabolic dysfunction-associated steatohepatitis (MASH) and advanced liver disease including fibrosis, cirrhosis and hepatocellular carcinoma [6, 7]. Patients with MASLD, especially those with MASH and hepatic fibrosis, are confronted with an excess risk of developing type 2 diabetes mellitus (T2DM), cardiovascular disease, chronic kidney disease and extrahepatic malignancies [5, 8, 9]. The highest prevalence of MASLD is observed in people with obesity (75%) or T2DM (69%) [7, 10]. These strong associations have led to a 2020 proposal to emphasize metabolic risks in the diagnosis of hepatic steatosis and adopt the term metabolic dysfunction-associated fatty liver disease (MAFLD), instead of the previously used term non-alcoholic fatty liver disease (NAFLD) [11]. In 2023, in an international multisociety Delphi consensus statement, the terms NAFLD and non-alcoholic steatohepatitis (NASH) were changed into MASLD and MASH, respectively [12]. With regard to cause-specific mortality in patients with MASLD, fewer than 10% develop fatal hepatic complications and the major causes of death comprise cardiovascular disease and cancer [13].

Of note, there is substantial heterogeneity in MASLD pathophysiology, which may differentially affect its clinical progression, association with cardiometabolic diseases, and response to treatment [14]. Some patients with MASLD have a stronger hepatic genetic component. Some others are characterized by a dominant metabolic component related to hepatic de novo lipogenesis in the setting of high glucose and fructose dietary intake and diabetes-related hyperglycemia and hyperinsulinemia. An additional subgroup is characterized by a dominant metabolic component related to adipose tissue dysfunction (lipodystrophy, visceral obesity, adipose tissue inflammation, dysregulated adipokine secretion) [14]. All these distinct MASLD phenotypes are associated with different risks of complications and rates of progression to fibrosis, which underscores the need for different and individualized treatment approaches.

Both obesity and metabolic disorders are tightly associated with MASLD [15, 16]. However, these two entities, although closely interlinked, do not always and invariably coexist. Several observational studies have shown that there is a subgroup of individuals with obesity who may be protected to some extent from obesity-related cardiometabolic complications and have significantly lower risk than would be expected based on the strong positive association between body mass index (BMI) and cardiometabolic risk [17]. This subset of obesity has been described as metabolically healthy obesity (MHO) and is characterized by the absence of cardiometabolic risk factors such as insulin resistance, impaired glucose metabolism, hypertension and dyslipidemia, despite excessive body fat accumulation [18–22]. MHO is an obesity phenotype with intermediate health risks falling between metabolically healthy non-obese (MHNO) individuals, typically used as the reference group, and subjects with metabolically unhealthy obesity (MUO), namely subjects with obesity and concomitant features of the metabolic syndrome (MS). Furthermore, not all individuals with obesity will develop MASLD or progress to hepatic fibrosis. Although it is widely recognized that the combined presence of abdominal obesity with T2DM may accelerate dramatically MASLD progression and increase significantly the risk of poor liver-related outcomes [3, 9, 10], the natural course and prognosis of MASLD in the MHO subgroup has not been adequately explored, and the hepatic health of subjects with MHO warrants further investigation. The actual risk of MASLD development and progression and the associated hepatic risks remain incompletely understood in the population with MHO. Based on current evidence, the MHO phenotype is associated with an increased risk of MASLD and progression to advanced stages when compared to MHNO individuals, but this risk is lower compared to patients with MUO, suggesting a strong synergistic interaction between obesity and metabolic risk factors in defining MASLD outcomes [23, 24].

The aim of the present narrative review is to summarize the existing evidence regarding the association between MHO and MASLD incidence, prevalence and progression. In this review, we address the controversial question of whether subjects with MHO have an increased risk of MASLD development and progression to hepatic fibrosis, and we also highlight the critical role of MASLD in predicting the conversion between different metabolic phenotypes of obesity. Furthermore, we critically discuss whether MASLD can be compatible with the MHO definition, and we emphasize the need to incorporate hepatic health assessment into the MHO concept, in order to improve its definition and predictive value for cardiometabolic outcomes.

## Literature Search and Review Criteria

For the preparation of this narrative review, we applied the search terms “metabolic-dysfunction associated steatotic liver disease”, “metabolic dysfunction-associated fatty liver disease”, “non-alcoholic fatty liver disease”, “fatty liver”, “hepatic fibrosis”, “obesity”, “metabolic health”, “obesity phenotypes”, “metabolically healthy obese”, “metabolically unhealthy obese”, in all possible combinations, in order to retrieve available literature data from PubMed, Scopus and Google Scholar from inception until April 2025. We included papers written in English language. We reviewed the reference lists of all manuscripts to detect additional relevant publications.

## Pathophysiological Concepts and Phenotypic Traits Associated with MHO

MHO is an intriguing concept based on the clinical observation that a subgroup of people with obesity do not exhibit clinically overt cardiometabolic abnormalities. The MHO phenotype is relatively common in clinical practice, prevalent in up to one third of individuals with obesity, with an age- and gender-dependent prevalence ranging between 10 and 30% [20, 25]. Overall, the prevalence of MHO appears to be higher in (premenopausal) women than men and in younger than older individuals [26]. A recent meta-analysis from 12 cohort and 7 intervention studies found a prevalence of MHO of 35% among people with obesity with significant regional variations [27]. There is still no standardized, universally accepted, definition for MHO. Despite the general consensus that a BMI ≥ 30 kg/m^2^ (≥ 25 for Asian and African populations) is required for the definition of MHO, more than 30 different definitions of metabolic heath have been applied in clinical studies [28], contributing to marked literature heterogeneity and complicating the interpretation of relevant study findings [18, 29, 30]. A harmonized definition of MHO has been proposed based on the diagnosis of obesity (BMI ≥ 30 kg/m^2^) and the fulfilment of all of the following criteria: serum triglycerides ≤ 1.7 mmol/l (≤ 150 mg/dl), HDL cholesterol > 1.0 mmol/l (40 mg/dl) (men) or > 1.3 mmol/l (50 mg/dl) (women), systolic blood pressure ≤ 130 mmHg, diastolic blood pressure ≤ 85 mmHg, no antihypertensive treatment, fasting blood glucose ≤ 5.6 mmol/l (≤ 100 mg/dl), and no glucose-lowering drug treatment [31]. The cardiovascular risk of subjects with MHO, when compared with MHNO individuals, is moderately elevated using the aforementioned widely used definitions of MHO [21]. However, when metabolic health is determined based on a recently proposed novel definition using risk factor and mortality data from the third National Health and Nutrition Examination Survey (NHANES III), including the absence of diabetes, no antihypertensive medications, systolic blood pressure < 130 mmHg and normal waist-to-hip ratio, MHO is not associated with elevated cardiovascular risk [21, 32].

Body composition and body fat distribution, including the ability to expand adipose tissue mass in the gluteofemoral fat compartment, play an important role in determining metabolic health of individuals with obesity [19, 21, 33]. In the first studies identifying and characterizing MHO and its underlying mechanisms in humans, it was suggested that liver fat is the predominant determinant of the insulin-sensitive phenotype of obesity, being even more relevant for insulin sensitivity than abdominal visceral fat [22]. In pathophysiological terms, subjects with MHO are characterized by reduced accumulation of intra-abdominal visceral and ectopic (including liver) fat for a given level of total fat, increased leg (gluteofemoral) fat deposition, less marked immune cell infiltration of visceral fat depots, lower adipocyte hypertrophy, enhanced expandability of subcutaneous adipose tissue, and preserved insulin sensitivity and pancreatic β-cell function. Furthermore, they exhibit normal adipose tissue function expressed as a favorable adipokine secretion profile, lower systemic and adipose tissue inflammation, a distinct pattern of circulating metabolites affecting whole-body insulin sensitivity, and higher cardiorespiratory fitness, when compared to subjects with MUO [20, 25, 34]. On the other hand, MUO, in direct analogy to human lipodystrophy, may be the result of an impaired ability of subcutaneous adipose tissue to further expand upon a chronic positive energy balance. Impaired adipose tissue function has been proposed as the critical mechanistic link between sustained energy imbalance and obesity-related end organ complications, including MASLD, diabetes and atherosclerotic cardiovascular disease [25].

From a clinical perspective, the view that MHO is a benign permanent phenotype of obesity, has been consistently challenged by data from large epidemiological studies and meta-analyses, clearly demonstrating that individuals with MHO are at a higher risk of cardiometabolic morbidity and mortality in comparison to MHNO [29, 35–38]. It appears that MHO is a transient obesity phenotype with moderate risk of subclinical metabolic dysfunction, which can convert into and from MUO during the natural course of obesity as well as in response to obesity treatment [21].

## MHO Is Associated with an Increased Risk of MASLD Development

Table 1 summarizes the key findings of the major cross-sectional and longitudinal cohort studies that investigated MASLD prevalence/incidence as well as progression in individuals with MHO [39–51]. In the majority of these studies, metabolic health has been defined as the absence or the presence of fewer than 2 components of the MS, and in some of them by applying even stricter metabolic criteria, as the combined absence of all MS components and lack of insulin resistance.

**Table 1:** An overview of the major cross-sectional and longitudinal cohort studies that have investigated MASLD prevalence and progression in subjects with MHO in descending order of publication year

First author, year of publication [ref]	Study design and study population	MHO definition	MASLD assessment	Key findings
Najafi F et al., 2024 [41]	Cross-sectional, population-based cohort study *N* = 8,360 adults	BMI ≥ 30 kg/m^2^ and absence of MS criteria	FLI ≥ 60 (based on triglycerides, BMI, GGT and WC)	OR for MASLD in males (vs. MHNO): - MHO: 8.92 (95% CIs 2.2–15.3) - MUNO: 7.23 (95% CIs 5.82–8.99) - MUO: 32.97 (95% CIs 15.70-69.22) Similar trends in females
Man S et al., 2022 [40]	Longitudinal health check-up cohort study *N* = 31,010 Chinese adults without MASLD at baseline Median follow-up: 2.2 years	BMI ≥ 25 kg/m^2^ and WC ≥ 80 cm (females) or ≥ 85 cm (males) and absence of MS components	Abdominal ultrasound and non-invasive fibrosis score FIB-4	HR for MASLD development in MHO (vs. MHNO): 5.51 (95% CIs 4.98–6.09) Similar pattern for the association between MHO and fibrosis progression
Tutunchi H et al., 2021 [47]	Cross-sectional study *N* = 246 Iranian patients with MASLD and low risk of fibrosis	BMI ≥ 25 kg/m^2^ and absence of MS components and HOMA-IR < 2.5	Abdominal ultrasound, NAFLD fibrosis score	Multi-variable adjusted OR for worsening of fibrosis in MHO: - BMI ≥ 30: 3.96 (95% CIs 2.89–4.71) - BMI 25-29.9: 1.99 (95% CIs 1.49–2.63) Excess body fat and WC were significantly associated with worsening hepatic fibrosis, regardless of metabolic health status
Chen T et al., 2021 [42]	Community-based population study *N* = 1,651 Asian subjects (Taiwan)	BMI ≥ 25 kg/m^2^ and < 2 MS components	Abdominal ultrasound semiquantitative measurements FLI 2–4: mild MASLD FLI ≥ 5: moderate-severe MASLD	OR for more severe MASLD after adjustment for visceral fat measured by BIA and other variables (vs. MHNO): - MHO: 2.44 (95% CIs 1.64–3.65) - MUNO: 2.75 (95% CIs 1.91–3.94) - MUO: 7.41 (95% CIs 4.94–11.12) Both obesity and metabolically unhealthy status were associated with increased MASLD risk, independent of visceral fat levels
Vusirikala A et al., 2020 [43]	Retrospective, population-based, longitudinal cohort study using The Health Improvement Network (THIN) database in United Kingdom *N* = 4,121,049 primary care patient records without baseline MASLD Median follow-up: 4.7 years	BMI ≥ 30 kg/m^2^ and absence of diabetes, hypertension and dyslipidemia	MASLD identified by Read Codes	Adjusted HR for incident MASLD in MHO (vs. MHNO): 6.92 (95% CIs 6.40–7.48) Metabolic risk factors further augmented this risk
Kim Y et al., 2019 [46]	Longitudinal cohort study *N* = 59,957 South Korean adults with MASLD Median follow-up: 7.7 years	BMI ≥ 25 kg/m^2^ and absence of MS components and HOMA-IR < 2.5	Abdominal ultrasound and NAFLD fibrosis score	Obesity was associated with worsening of NAFLD fibrosis score, regardless of metabolic abnormalities
Ampuero J et al., 2018 [49]	Cross-sectional study *N* = 1,058 Spanish patients with biopsy-proven MASLD	BMI > 30 kg/m^2^ and absence of MS components	Liver biopsy	Both MHO and MUO were independently associated with an increased risk of MASH and significant hepatic fibrosis
Haskins IN et al., 2018 [51]	Cross-sectional cohort study *N* = 270 obese patients undergoing bariatric surgery in a tertiary academic referral hospital in USA (2008–2015)	BMI > 30 kg/m^2^ and absence of hypertension, dyslipidemia, prediabetes/diabetes before surgery	Intra-operative liver biopsy	35.5% had MASLD with variable NAFLD activity scores − 23% had lobular inflammation − 8.5% had hepatocyte ballooning − 8.2% had MASH − 4.4% had hepatic fibrosis
Gutiérrez-Grobe Y et al., 2017 [48]	Cross-sectional study *N* = 428 adults with MASLD	BMI > 30 kg/m^2^ and absence of MS components	Abdominal ultrasound, transient elastography, NAFLD score, APRI	MHO had a lower prevalence of advanced fibrosis (F3-F4) compared to MUO (16.5% vs. 28.0%, *p* ≤ 0.05)
Huh JH et al., 2017 [45]	Cross-sectional study *N* = 2,198 Korean adults undergoing a medical health check-up	BMI ≥ 25 kg/m^2^ and absence of MS components	Abdominal ultrasound and vibration-controlled transient elastography	MHO was associated with an increased prevalence of MASLD and advanced fibrosis OR for hepatic steatosis (vs. MHNO): - MHO: 4.62 (95% CIs 3.52–6.07) - MUNO: 2.94 (95% CIs 2.32–3.71) - MUO: 12.02 (95% CIs 9.08–15.92) No increased fibrosis risk in MUNO, but increased risk in MHO OR 4.32 (95% CIs 1.73–10.76) in the fully adjusted model
Chang Y et al., 2016 [39]	Longitudinal cohort study *N* = 77,425 South Korean adults without baseline MASLD, participating in a health screening examination program Median follow-up: 4.5 years	BMI ≥ 25 kg/m^2^ and absence of MS components and HOMA-IR < 2.5	Abdominal ultrasound	BMI categories were progressively associated with an increased MASLD incidence Multi-variable adjusted HR for incident MASLD in MHO vs. MHNO: 3.55 (95% CIs 3.37–3.74)
Lee M et al., 2015 [44]	Retrospective study *N* = 3,045 Korean healthy subjects without diabetes or MASLD at baseline Follow-up: 4 years	BMI > 25 kg/m^2^ and < 2 MS components	Abdominal ultrasound	OR for MASLD (vs. MHNO): - MHO: 1.731 - MUNO: 1.877 - MUO: 2.501 MASLD risk was significantly higher in MUNO than MHO
Sung K et al., 2014 [49]	Cross-sectional occupational cohort study *N* = 14,384 South Korean adults	BMI ≥ 25 kg/m^2^ and WC ≥ 80 cm (females) or ≥ 90 cm (males) and absence of MS components beyond obesity	Abdominal ultrasound	Prevalence of MASLD in MHO: 45% OR for MASLD (vs. MHNO): - MHO: 3.63 (95% CIs 3.06–4.31) - MUNO: 1.83 (95% CIs 1.69–2.08) - MUO: 5.89 (95% CIs 5.18–6.70)

APRI: AST-to-platelet ratio index; BIA: bioelectrical impedance analysis; BMI: body mass index; CIs: confidence intervals; FIB-4: fibrosis risk score 4; FLI: fatty liver index; GGT: gamma glutamyl transferase; HOMA-IR: homeostasis model assessment index of insulin resistance; HR: hazard ratio; MASH: metabolic dysfunction-associated steatohepatitis; MASLD: metabolic dysfunction-associated steatotic liver disease; MHNO: metabolically healthy non-obese; MHO: metabolically healthy obese; MS: metabolic syndrome; MUNO: metabolically unhealthy non-obese; MUO: metabolically unhealthy obese; NAFLD: non-alcoholic fatty liver disease; OR: odds ratio; USA: United States of America; WC: waist circumference

In a large-scale longitudinal cohort study of 77,425 South Korean individuals, who were strictly defined as being metabolically healthy by having no MS components, no ultrasound-defined MASLD at baseline and a homeostasis model assessment index of insulin resistance (HOMA-IR) below 2.5, increasing baseline BMI was found to be independently associated with an increased incidence of MASLD over a mean follow-up period of 4.5 years, suggesting that obesity per se, regardless of the coexistence of metabolic abnormalities, can significantly increase the risk of developing MASLD [47]. The detailed dose-response analyses of this study clearly suggested that increasing BMI had a strong and almost linear relationship with MASLD incidence [47]. Of note, this strong progressive association between BMI and MASLD was more pronounced in women, observed across the whole range of BMI, and confirmed in all subgroups evaluated, including participants with low inflammation status [47].

In the same direction, an additional longitudinal health check-up cohort study in 31,010 Chinese adults without MASLD at baseline, has shown that the MHO phenotype is associated with significantly higher risks of MASLD and fibrosis progression over a median follow up period of 2.2 years compared to MHNO subjects, suggesting that, in terms of MASLD prevention, subjects with MHO are expected to gain substantial benefits from weight loss interventions targeting excess adiposity, independent of concurrent metabolic abnormalities [40]. Of note, the association between increasing BMI and MASLD risk was found to be exponential, suggesting that as BMI gradually increased, the associated MASLD risk increased more rapidly [40]. Additional interesting findings were a stronger association between MHO and MASLD in females than males, and a stronger association between MHO and advanced MASLD with fibrosis progression in younger (< 50 years old) compared to older patients [40].

Several studies, comparing MASLD prevalence among different metabolic subgroups of people with and without obesity (MHO, MUO, MHNO, MUNO), have shown that both obesity and metabolic disorders may independently contribute to an increased MASLD risk, in the context of a synergistic dose-response relationship. In a cross-sectional, population-based analysis of 8,360 Iranian adults, it was recently shown that the MASLD prevalence in males increased by 8.92 times in MHO, 7.23 times in MUNO, and 32.97 times in MUO, compared to the MHNO reference group, and similar trends were reported for females [39]. In another community-based population study in 1,651 Asian subjects, it was shown that both obesity and an unhealthy metabolic status pose an increased MASLD risk, independent of visceral fat levels, but this risk was the highest in the MUO group, suggesting a synergistic effect of obesity with metabolic abnormalities on MASLD risk [42]. These findings are in agreement with a previous retrospective, population-based, longitudinal cohort study using > 4 million patient records from a United Kingdom primary care database, which provided the first evidence in a European population that MHO is associated with an increased risk of MASLD incidence over a median follow up period of 4.7 years, and metabolic risk factors such as diabetes, hypertension and dyslipidemia further potentiate this risk [43]. Although there is robust evidence that MASLD risk is the highest in subjects with MUO, whether this risk is higher in the MHO or MUNO group, namely whether metabolic health or rather obesity is relatively more important for MASLD development, remains debatable. On the one hand, some data have shown that being metabolically unhealthy may contribute more to MASLD than obesity [48]. On the other hand, it has been shown that obesity, even in the absence of metabolic risk factors, is more closely associated with hepatic steatosis and fibrosis than an unhealthy metabolic status with normal bodyweight [50].

## MHO Is Associated with an Increased Risk of MASLD Progression

MASLD progression has been mainly assessed by means of non-invasive fibrosis indices (i.e. liver stiffness measurement obtained by transient elastography or fibrosis risk scores derived from anthropometric, clinical and biochemical parameters assessed in blood laboratory measurements) in most relevant studies.

In a large-scale cohort study of nearly 60,000 Korean adults with ultrasound-defined MASLD, BMI was independently associated with progressive worsening of liver fibrosis, being non-invasively assessed by the NAFLD fibrosis score, in both subjects with MHO and MUO over a median follow-up of 7.7 years [44]. These longitudinal data were further corroborated by the findings of a cross-sectional analysis of 246 Iranian patients with MASLD, which demonstrated that increasing BMI is associated with more severe hepatic fibrosis, assessed non-invasively by NAFLD fibrosis score, in both metabolically healthy and unhealthy individuals, indicating that excess body fat accumulation may independently promote the progression of MASLD to fibrosis regardless of metabolic health status [41].

Although there is clear evidence that subjects with MHO can progress to advanced MASLD despite their apparently normal metabolic health profile [44, 50], the prevalence of MASH and clinically significant liver fibrosis appears to be lower in MHO compared to MUO [45, 46]. In a Spanish study of nearly 1,100 patients with biopsy-confirmed MASLD, the risk of having MASH was found to be nearly 2-fold elevated in subjects with MHO compared to non-obese individuals, but this risk was even higher (nearly 3.5-fold increased) in MUO, after adjustment for several variables related to insulin resistance and liver biochemistry [45]. Similar findings were reported for the risk of significant hepatic fibrosis [45].

## Underlying Mechanisms and Factors Modifying the Relationship Between MHO and MASLD

The synergistic detrimental effect of the combined presence of obesity and metabolic abnormalities on MASLD outcomes can be explained by the traditional “two-hit” theory which has been proposed to apply for MASLD pathogenesis. The “first hit” is considered to be excess adiposity, leading to increased free fatty acid release and influx to the liver resulting in hepatic steatosis. Hepatic fat may in turn sensitize the liver to the “second hit”. Metabolic abnormalities may provoke the “second hits”, mainly comprising inflammation and oxidative stress, which further promote the progression of MASLD to MASH and fibrosis [52].

Hyperuricemia has been proposed as an effect modifier of the association between different metabolic phenotypes of obesity and MASLD risk. A population-based, cross-sectional study in nearly 3,000 Chinese adults provided evidence for a synergistic interaction between elevated serum uric acid levels and MHO in augmenting the risk of having MASLD [53]. According to this study, MASLD prevalence was significantly higher in participants with MHO and hyperuricemia compared to MHO with normal uric acid levels, suggesting that serum uric acid levels, often viewed as a surrogate of insulin resistance, may modify the association between MHO and MASLD [53].

Genetic factors may also affect the relationship between MHO and MASLD, considering the reported interaction effects between specific MASLD-related genetic polymorphisms, metabolic status and obesity. The most commonly studied genes which are thought to predispose to MASLD and can be synergistically affected by concurrent obesity, are PNPLA3 and TM6SF2, which are involved in the hepatic production of triglyceride-rich lipoproteins [54]. In this context, a study applying RNA sequencing in liver and visceral fat samples obtained intra-operatively from patients with obesity undergoing bariatric surgery, identified a unique transcriptomic signature which could diagnose MASLD and discriminate between MHO and MUO phenotypes [55]. This study found an upregulation of genes related to inflammation and lipid metabolism and a down-regulation of genes involved in insulin signaling and mitochondrial function (oxidative phosphorylation) in both liver and visceral fat tissue of patients with MUO and MASLD compared with MHO, indicating different transcriptional profiles between MHO and MUO in promoting MASLD [55].

## The Presence of MASLD May Predict the Phenotypic Conversion from MHO To MUO

**Fig. 1:**
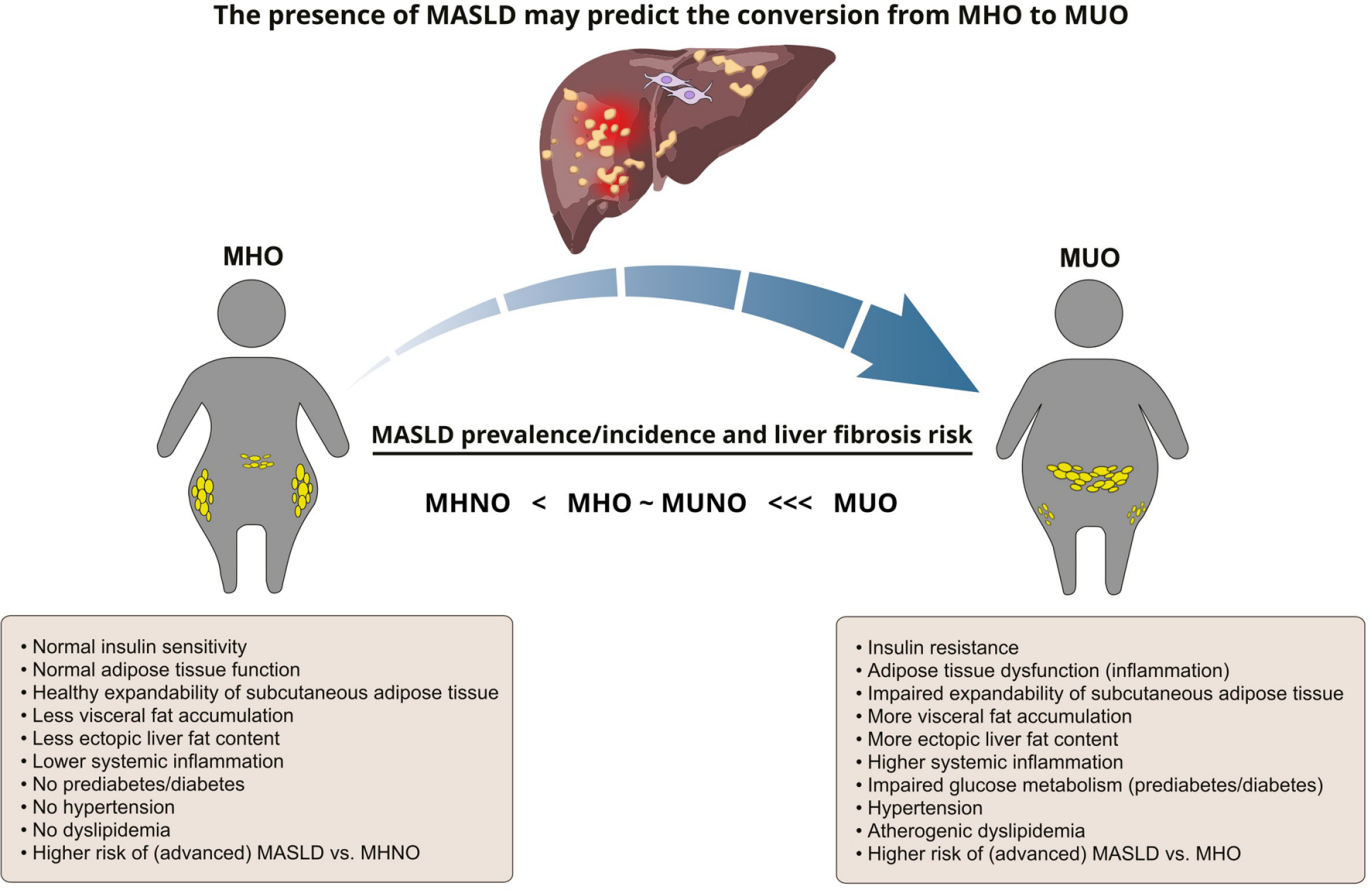
The presence of MASLD may predict the conversion from the MHO to the MUO phenotype. MASLD prevalence and hepatic fibrosis risk are elevated both in MHO and MUNO, compared to the MHNO reference group, but these risks are significantly lower compared to the MUO group, suggesting a strong synergistic adverse interaction between obesity and metabolic risk factors in defining MASLD outcomes. MASLD: metabolic dysfunction-associated steatotic liver disease; MHO: metabolically healthy obese; MUO: metabolically unhealthy obese; MUNO: metabolically unhealthy non-obese; MHNO: metabolically healthy non-obese

Metabolic health is not a static but rather a dynamic condition, which may fluctuate over a long-term period. It has been reported that 33–52% of individuals with increased bodyweight may convert from the MHO to the MUO phenotype over a follow-up period of 6–20 years [56–62]. This transition rate varies considerably in various studies due to regional ethnic-specific differences, different duration of follow-up and heterogeneous diagnostic criteria for defining obesity and metabolic health. It has been suggested that systemic inflammation, oxidative stress, gut microbiota alterations and genetic risk factors may be pathogenetically involved in this conversion [63]. In this context, the baseline presence of MASLD has emerged as a critical factor with independent predictive value for the conversion from the MHO to the MUO phenotype (Fig. 1).

A prospective, population-based, observational cohort study in a large Greek sample of the greater metropolitan Athens area (ATTICA study) in a total of 3,042 participants, investigated the role of baseline ultrasound-defined MASLD in predicting the transition from the MHO to the MUO phenotype over a period of 10 years [64]. In this study, subjects with MHO, defined as having BMI ≥ 30 kg/m^2^ and absence of all MS criteria, who became metabolically unhealthy over the 10-year follow-up period, had about two times higher odds to have MASLD at baseline compared with their MHNO counterparts, and furthermore, subjects with MHO and baseline MASLD displayed an elevated 10-year cardiovascular risk compared to MHO without baseline MASLD [64]. These data clearly demonstrate that MASLD may contribute independently and accurately to determine future cardiometabolic risk in individuals with obesity.

Similar data have been provided by other investigators as well. In a Chinese prospective study in a large population of 12,910 participants who were defined as metabolically healthy based on ≤ 1 MS components, 27% of subjects converted into the unhealthy phenotype over a median follow-up period of 6.9 years, and baseline MASLD (determined by abdominal ultrasound) was found to be a strong significant predictor of conversion, independently of potential confounders, both in the group with and without obesity [65]. An interesting finding of this study was that the effect of MASLD on future metabolic health deterioration was stronger in the lean than in the overweight and obese group, suggesting that the lean MASLD phenotype is not benign at all and requires intensive long-term metabolic monitoring [65]. These results are in line with the findings of another South Korean cohort study in a total of 22,551 metabolically healthy individuals [66]. In the latter study, 23.5% of the total population converted into the unhealthy phenotype over a median follow-up period of 5.1 years, and baseline MASLD (assessed by ultrasound) was able to predict this conversion, independent of age, sex, BMI, lifestyle factors, MS components and insulin resistance. Notably, as in the Chinese study [65], the impact of MASLD on metabolic deterioration differed according to obesity severity, and weakened as BMI and fat mass increased [66].

On the other hand, the presence of MASLD has been identified as a strong independent predictor of failure to restore metabolic health and convert from the MUO to the MHO phenotype in the setting of a weight loss lifestyle intervention [67], suggesting that hepatic steatosis is a key element for the transition between different metabolic phenotypes of obesity in either direction.

With regard to the underlying pathophysiological mechanisms which can explain the critical role of MASLD in inducing and accelerating metabolic health deterioration, it has been suggested that MASLD can exacerbate both hepatic and systemic (whole-body) insulin resistance, predispose to atherogenic dyslipidemia and trigger the release of several proinflammatory and vasoactive mediators that may promote the pathogenesis and clinical manifestation of various obesity-associated cardiometabolic complications [68]. In the setting of MASLD, the inflamed steatotic liver may affect the heart and vasculature via increased hepatic glucose production, release of procoagulant and proatherogenic factors, and dysregulated secretion of hepatokines. All these pathophysiological alterations may pave the way for the establishment of clinically relevant cardiovascular and metabolic complications.

## Impact of Weight Loss on MASLD Outcomes in MHO

Clinical practice guidelines for MASLD management recommend a weight loss of at least 5% to improve hepatic steatosis, and an even greater weight loss, equal to 10%, to increase the likelihood of resolution and potential regression of hepatic inflammation and fibrosis [2, 69]. Although lifestyle modification to achieve clinically meaningful weight loss has proven beneficial effects on MASLD outcomes and is considered to be a first-line intervention for patients with established MASLD [70, 71], relatively little is known about whether subjects with MHO may benefit from the effects of weight loss in terms of reducing their risk of developing (advanced) MASLD. Current evidence suggests that weight loss can produce substantial benefits in preventing MASLD development and reducing the risk of advanced MASLD with hepatic fibrosis in subjects with MHO. In a longitudinal retrospective cohort study in nearly 15,000 participants, investigating the impact of weight change of different magnitude on MASLD incidence and fibrosis risk, it was reported that a clinically relevant weight loss of 5% was associated with a reduced risk of developing MASLD with or without risk of advanced liver fibrosis as assessed by two non-invasive fibrosis risk scores, in metabolically healthy overweight/obese Korean individuals (BMI ≥ 23 kg/m^2^) [72]. In this study, metabolic health was defined as the absence of MS components and HOMA-IR < 2.5. The study has shown that those subjects who managed to achieve a weight loss of more than 5%, reduced their risk of future MASLD development by nearly 50% over a median follow up period of 5.2 years, while those who achieved a less pronounced weight loss ranging between 1 and 5%, reduced their respective risk by 17%, compared to the no weight change group [72]. Of note, in mediation analyses, further adjustment for insulin resistance, inflammation and other metabolic components did not significantly alter the observed strong associations between weight change, MASLD development and fibrosis risk [72]. These data were further confirmed by another longitudinal study of similar design in a Chinese population of 5,625 subjects with overweight/obesity who were defined as metabolically healthy by not having any MS components, which found that those subjects who achieved a weight loss of more than 5% reduced their risk of MASLD development by 64% over a median follow up period of 2.1 years, and those who achieved a weight loss of 1–5% reduced also their risk by 41% [73]. The most interesting finding of the latter study was the more marked gradient of cumulative MASLD incidence across weight change categories observed in MHO compared to MUO, suggesting a more evident association between weight loss and MASLD risk in MHO, and strengthening thus the notion that weight loss interventions targeting individuals with MHO might have greater public health benefits in MASLD prevention compared to targeting MUO [73].

## MHO with MASLD: Can this Phenotype Truly Exist??

MHO with MASLD is not the same as MHO without MASLD in terms of overall cardiometabolic risk. Although a study has provocatively suggested that more than a quarter (26.6%) of MASLD patients can be metabolically healthy, defined as not having diabetes and having < 2 metabolic abnormalities, and further reported that this subgroup is characterized by similar hepatic fibrosis burden and atherosclerotic risk as the healthy control group [74], the prevalent notion is that MHO with MASLD cannot be viewed as truly metabolically healthy [75]. A Chinese cohort study has shown that compared with MHO without MASLD, subjects with MHO and MASLD display an excessive risk of prediabetes and diabetes, highlighting the need to screen all subjects with MHO for the concomitant presence of MASLD [76]. A meta-analysis of prospective cohort studies has also shown that MHO with MASLD may increase three-fold the relative risk of incident diabetes [77]. These data, combined with the fact that the presence of MASLD has been proposed as a risk factor for developing metabolic abnormalities and major adverse cardiovascular events in the subsequent years [64–66], as previously discussed, emphasize the need to incorporate hepatic health assessment into the MHO concept in order to improve its definition and predictive utility for cardiometabolic outcomes. Taking all evidence into consideration, MASLD represents an early predictor of metabolic dysfunction and increased cardiovascular risk in subjects currently classified as MHO, and should be always evaluated in order to delineate metabolic health and classify the population with obesity into MHO and MUO. Actually, its presence should be used as an exclusion criterion for the diagnosis of the MHO phenotype.

## Summary of Key Findings

Both obesity and metabolic disorders are strongly associated with MASLD. However, not all individuals with obesity will develop MASLD or progress to hepatic fibrosis. Although the combination of obesity with metabolic risk factors is known to accelerate MASLD progression and increase the risk of poor hepatic outcomes, the natural course of MASLD in the MHO subgroup has not been adequately explored.

Several studies comparing MASLD prevalence and incidence among different metabolic phenotypes of obesity, have shown that both obesity and metabolic disorders may independently contribute to an increased MASLD risk, in the context of a synergistic relationship. Based on a large number of cross-sectional and longitudinal cohort studies, the MHO phenotype is associated with an increased risk of MASLD and progression to fibrosis when compared to MHNO individuals, but this risk is lower compared to patients with MUO. Furthermore, although there is clear evidence that subjects with MHO can progress to advanced MASLD despite their apparently normal metabolic health profile, the prevalence of MASH and significant liver fibrosis appears to be lower in MHO compared to MUO, suggesting a strong synergistic interaction between obesity and metabolic risk factors in defining MASLD outcomes.

MASLD has emerged as a critical factor with independent predictive value for the conversion from the MHO to the MUO phenotype. In addition, the presence of MASLD has been identified as an independent predictor of failure to restore metabolic health and convert from the MUO to the MHO phenotype in the setting of a weight loss intervention, suggesting that hepatic steatosis is a key element for the transition between different metabolic phenotypes of obesity in either direction. Beyond metabolic health deterioration, it has been further suggested that the presence of MASLD can increase the risk of major adverse cardiovascular events in the MHO population. All these data emphasize the need to incorporate hepatic health assessment into the MHO concept, in order to refine its definition and predictive utility for cardiometabolic outcomes.

## Challenges, Controversies and Clinical Implications

The hepatic health of subjects with MHO warrants further investigation. Additional research is needed to better understand the role of MASLD in mediating the conversion from MHO to MUO and elucidate how this phenotypic transition may affect MASLD clinical outcomes. More research is also needed to address the question of whether and to what extent subjects with MHO may benefit from the effects of weight loss in terms of reducing their risk of developing (advanced) MASLD.

An open, still unresolved, issue remains whether metabolic health or rather obesity is relatively more important for MASLD development. On the one hand, some data have shown that being metabolically unhealthy may contribute more to MASLD than obesity per se. On the other hand, it has been shown that obesity, even in the absence of metabolic risk factors, is more closely associated with hepatic steatosis and fibrosis than an unhealthy metabolic status with normal bodyweight.

Whether the phenotype of MHO with concomitant hepatic steatosis can truly exist remains also controversial. The finding that subjects with MHO and MASLD display an increased risk of prediabetes and diabetes, compared with MHO without MASLD, underscores the need to screen all subjects with MHO for the concomitant presence of MASLD. In this respect, the reduced diagnostic accuracy of liver steatosis and fibrosis quantification by means of ultrasound and transient elastography in patients with severe obesity remains a clinical challenge, and highlights the need for a wider implementation of non-invasive fibrosis risk scores in this population. Based on the findings that subjects with MHO and MASLD, even in the absence of diabetes, hypertension or dyslipidemia, have excess risk of developing metabolic abnormalities, cardiovascular disease and advanced hepatic fibrosis in the subsequent years, clinicians should screen all subjects with MHO for the presence of MASLD by means of abdominal ultrasound and non-invasive fibrosis risk stratification scores. The presence of MASLD in these apparently metabolically healthy individuals would signify an elevated risk for adverse hepatic and cardiometabolic outcomes and warrant intensive metabolic monitoring and structured weight loss interventions. In this context, subjects with MHO are expected to gain substantial benefits from weight loss interventions targeting excess adiposity, independent of concurrent metabolic abnormalities. Furthermore, the more evident association between weight loss and MASLD risk in MHO vs. MUO, strengthens the notion that weight loss interventions targeting subjects with MHO might have greater public health benefits in MASLD prevention compared to targeting MUO.

## Conclusion

In conclusion, MASLD represents an early predictor of metabolic dysfunction and increased cardiovascular risk in subjects who are currently classified as MHO, and should be always assessed in order to fully characterize metabolic health and classify obesity into MHO and MUO. We suggest that the presence of MASLD should be used as an exclusion criterion for the diagnosis of the MHO phenotype.

## Key References

- Schulze MB, Stefan N. Metabolically healthy obesity: from epidemiology and mechanisms to clinical implications. Nat Rev Endocrinol. 2024;20:633–46. 10.1038/s41574-024-01008-5
-
  - This is a state-of-the-art review regarding epidemiology, pathophysiological mechanisms and clinical implications of metabolically healthy obesity.
- Lonardo A, Mantovani A, Lugari S, Targher G. Epidemiology and pathophysiology of the association between NAFLD and metabolically healthy or metabolically unhealthy obesity. Ann Hepatol. 2020;19:359–66. 10.1016/j.aohep.2020.03.001
-
  - This review addresses the epidemiology and pathophysiological links between NAFLD and metabolically healthy or unhealthy obesity.
- Man S, Lv J, Yu C, Deng Y, Yin J, Wang B, et al. Association between metabolically healthy obesity and non-alcoholic fatty liver disease. Hepatol Int. 2022;16:1412–23. 10.1007/s12072-022-10395-8.
-
  - This cohort study investigated the association between metabolically healthy obesity and NAFLD, and concluded that MHO is associated with significantly higher risks of NAFLD and fibrosis progression.
- Tutunchi H, Naeini F, Ebrahimi-Mameghani M, Najafipour F, Mobasseri M, Ostadrahimi A. Metabolically healthy and unhealthy obesity and the progression of liver fibrosis: A cross-sectional study. Clin Res Hepatol Gastroenterol. 2021;45:101754. 10.1016/j.clinre.2021.101754
-
  - This cross-sectional study aimed to examine the association of liver fibrosis with metabolically healthy and unhealthy obesity among patients with NAFLD, and concluded that excess body fat contributes to the progression of liver fibrosis regardless of metabolic health status.
- Kim Y, Chang Y, Cho YK, Ahn J, Shin H, Ryu S. Metabolically healthy versus unhealthy obesity and risk of fibrosis progression in non-alcoholic fatty liver disease. Liver Int. 2019;39:1884–94. 10.1111/liv.14184
-
  - This study investigated the association of obesity with worsening of non-invasive liver fibrosis markers in metabolically healthy and unhealthy individuals with NAFLD. It found that BMI is positively associated with worsening of fibrosis regardless of metabolic health status, and suggested that excess adiposity per se, even without accompanying metabolic abnormalities, may contribute to fibrosis progression in NAFLD.
- Chang Y, Jung HS, Cho J, Zhang Y, Yun KE, Lazo M, et al. Metabolically healthy obesity and the development of nonalcoholic fatty liver disease. Am J Gastroenterol. 2016;111:1133–40. 10.1038/ajg.2016.178
-
  - This study examined the association between body mass index categories and the development of NAFLD in a large cohort of metabolically healthy subjects, and concluded that obesity is strongly and progressively associated with an increased NAFLD incidence, regardless of concurrent metabolic abnormalities.
- Kouvari M, Chrysohoou C, Skoumas J, Pitsavos C, Panagiotakos DB, Mantzoros CS. The presence of NAFLD influences the transition of metabolically healthy to metabolically unhealthy obesity and the ten-year cardiovascular disease risk: A population-based cohort study. Metabolism 2022:128:154893. 10.1016/j.metabol.2021.154893
-
  - This population-based prospective cohort study reported that the baseline presence of NAFLD can predict the conversion from the MHO to the MUO phenotype and increase the 10-year cardiovascular disease risk, suggesting that liver steatosis may contribute independently and accurately to determine future cardiometabolic risk in individuals with obesity.

## Data Availability

No datasets were generated or analysed during the current study.

## Declarations

### Short Running Title

Metabolically healthy obesity and MASLD.
